# Undifferentiated carcinoma with osteoclast-like giant cells of the pancreas diagnosed by endoscopic ultrasound guided biopsy

**DOI:** 10.3332/ecancer.2020.1072

**Published:** 2020-07-17

**Authors:** Daniela Speisky, Mariano Villarroel, Félix Vigovich, Alejandro Iotti, Teresa Adriana García, Luciana Bella Quero, Mariano Bregante, Maria Teresa García de Dávila

**Affiliations:** 1 Department of Histopathology, Hospital Británico, Buenos Aires C1280AEB, Argentina; 2 Department of Gastroenterology, Digestive Endoscopy Section, Hospital Británico, Buenos Aires C1280AEB, Argentina; 3 Department of Diagnostic Imaging, Hospital Británico, Buenos Aires C1280AEB, Argentina; 4 Department of Oncology, Hospital Británico, Buenos Aires C1280AEB, Argentina; 5 Department of General Surgery, Biliopancreatic Area, Hospital Británico, Buenos Aires C1280AEB, Argentina

**Keywords:** endoscopic ultrasound, giant cell tumours, undifferentiated carcinoma, osteoclastic giant cells, pancreas

## Abstract

Undifferentiated pancreatic carcinoma with osteoclast-like giant cells is a rare tumour that has been published under a wide variety of names, including pleomorphic carcinoma, giant cell carcinoma, sarcomatoid carcinoma and carcinosarcoma, among others. For these reasons and its low frequency, the reports of these tumours are scarce and frequently lead to confusion with other entities which present with giant cells.

We present the case of a patient with obstructive jaundice and a mixed cystic and solid pancreatic mass, accompanied by multiple hepatic lesions.

The histological study of the material obtained by endoscopic ultrasound guided biopsy demonstrated a proliferation of atypical epithelioid cells, accompanied by a spindle cell component with marked pleomorphism and numerous osteoclast-like giant cells. The epithelioid component showed positive immunostaining with cytokeratin cocktail and cytokeratin 7. The spindle cell component showed coexpression of cytokeratins and vimentin. The osteoclast-like giant cells were positive for CD68. Protein p53 was overexpressed in both epithelial and spindle cell neoplastic components, and was negative in the giant cells. These findings permitted the diagnosis of undifferentiated carcinoma of the pancreas with osteoclast-like giant cells. This case outlines the effectiveness of endoscopic ultrasound-guided biopsy and the importance of morphological and immunohistochemical examination in the diagnosis of different types of pancreatic tumours, especially when they are in advanced stages and are not suitable for surgical treatment.

## Introduction

Undifferentiated carcinoma with osteoclast-like giant cells (UC-OGC) of the pancreas is a rare tumour, which accounts for less than 1% of all pancreatic malignancies. However, its true incidence is not totally established due to the great variation in names under which it has been published, including: pleomorphic carcinoma, giant cell carcinoma, sarcomatoid carcinoma and carcinosarcoma, among others [1–3].

Morphologically, these tumours are defined by the presence of non-neoplastic osteoclast-like giant cells (OGC), which accompany the carcinomatous component of the tumour. Immunohistochemical, molecular and ultrastructure studies indicated that the giant cell component consists of reactive histocytes attracted by the neoplastic cells of the carcinoma [1]. Macroscopically, UC-OGC are characterised by their great size, being able to reach 5 to 10 cm at the time of diagnosis. Some can present with polypoid growth to the papilla or the main pancreatic duct, or show cystic degeneration [1, 4].

The histogenesis of this type of tumour is controversial. It was initially hypothesised that they could derive from acinar cells, mesenchymal cells or pluripotent precursor cells. However, their origin was actually established to be in epithelial ductal cells and was included as a variant of pancreatic ductal adenocarcinoma (PDAC) within the classification of the WHO [1, 5–7].

Our case highlights the effectiveness of endoscopic ultrasound (EUS) guided biopsy in histopathological studies of pancreatic tumours, especially in advanced stages not suitable for surgical treatment.

## Case report

A female patient aged 67 years, with a 1-year history of diabetes, consulted our institution for diarrhoea of 2-month duration, loss of weight (9 kg in 1 year) and generalised pruritus. The clinical examination presented jaundice of the skin and mucosae, acholia and choluria. On abdominal examination, there was an ill-defined mass in the right hypochondrium and epigastrium with hard tenderness.

**Figure 1.**
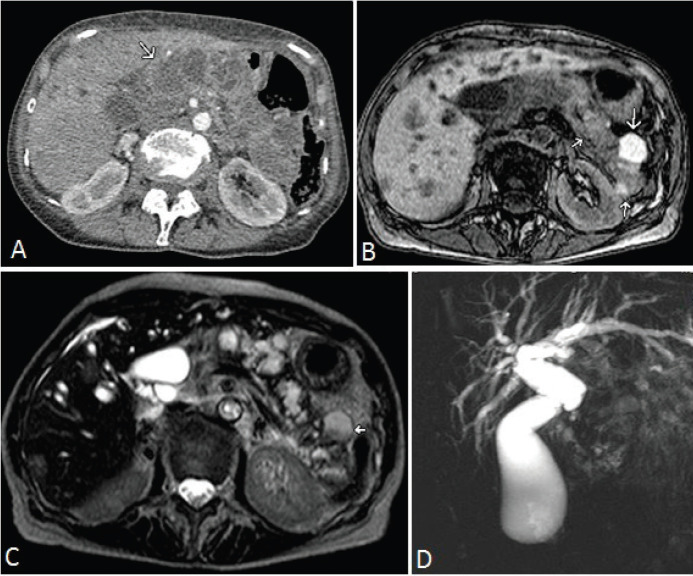
A) Arterial phase CT scan. Multiple cysts (arrow) which replace pancreatic parenchyma. B) Magnetic resonance imaging, T1 weighted sequence, without contrast demonstrates hypointense focal images and others of different sizes which are hyperintense (arrows). C) The T2 weighted sequence shows hyperintense cystic focal images and others which are heterogeneously hyper and hypointense consistent with the image descriptions in B (arrow). D) Cholangioresonance shows dilatation of the bile duct with a blockage in the intrapancreatic bile duct and the disappearance of the Wirsung duct by replacement of the pancreatic parenchyma by the tumour.

Laboratory tests revealed a cholestatic injury associated with a huge increase in Ca 19.9 (72,938 U/ml, normal value (NV): 3–27 U/ml) and a minor increase in CEA (14.8 ng/ml, NV: 1–5.2 ng/ml). The study of ultrasound, computed tomography (CT scan) and magnetic resonance imaging showed a voluminous lesion both solid and cystic in the head of the pancreas extending throughout the body and tail, accompanied by multiple hepatic images suggestive of secondaries (Figure 1A–C). The magnetic resonance cholangiopancreatography showed marked intra- and extrahepatic dilation of the bile duct in relation to the expansive formation described previously (Figure 1D).

**Figure 2.**
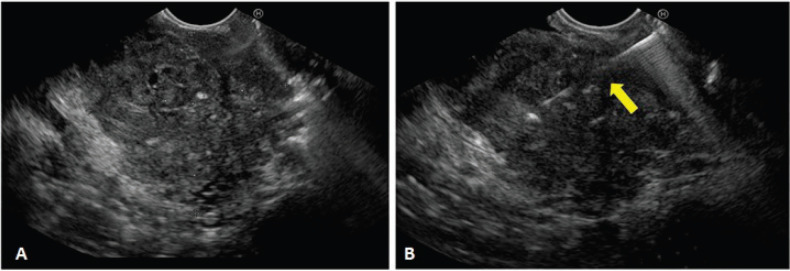
Endoscopic Ultrasound (EUS). A) Hypoechoic heterogeneous mass, with irregular borders, located in the head of the pancreas. B) Observe the distal end of the biopsy needle entering the tumour (yellow arrow).

A biliopancreatic EUS and an endoscopic retrograde cholangiopancreatography (ERCP) for biliary drainage were performed. On the EUS, a hypoechoic mass with heterogeneous echotexture was identified at the level of the head of the pancreas, measuring 52 × 50 mm in contact with the splenoportal axis and invasion of the distal bile duct (Figure 2). Fine needle aspiration biposy was performed using 22 G (Boston Scientific ®, Massachusetts, USA), and three samples were sent to pathology. The ERCP evidenced extrinsic compression of the duodenal bulb and a swollen papilla. Conventional cannulation was attempted without success. Therefore, a precut was performed, observing a polypoid lesion which was obstructing the bile duct. It was resected with a polypectomy snare and sent to pathology (video 1). After a few weeks, biliary drainage was achieved through a ‘percutaneous-endoscopic rendezvous procedure’. Once biliary cannulation had been achieved, a partially covered 10 × 80 mm self-expandable metallic stent was placed (Boston Scientific ®, Massachusetts, USA). The patient improved initially but later deteriorated physically and functionally. Consequently, symptomatic treatment was commenced, and she died 3 months after the diagnosis.

**Figure 3.**
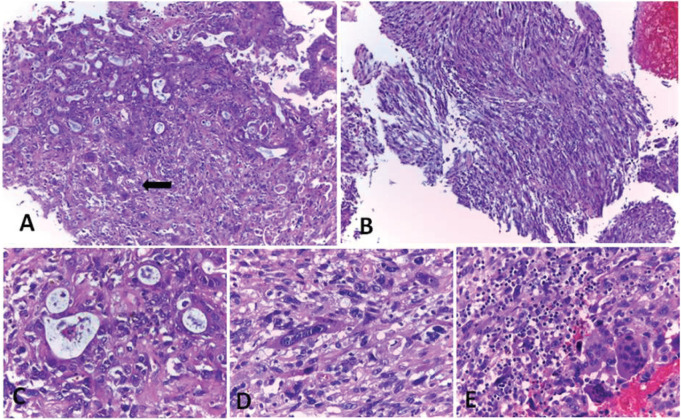
Undifferentiated carcinoma with osteoclast-like giant cells coloured with haematoxylin and eosin. A) Epithelioid component of the carcinoma with glandular pattern associated with OGC (arrow). B) Spindle cell component of the carcinoma. C, D and E) High magnification of A and B. C) Adenocarcinoma, D) Spindle cell component and E) OGC. Original magnification: 100× (A, B); 400× (C, D and E). Ref: OGC: osteoclast-like giant cells.

**Figure 4.**
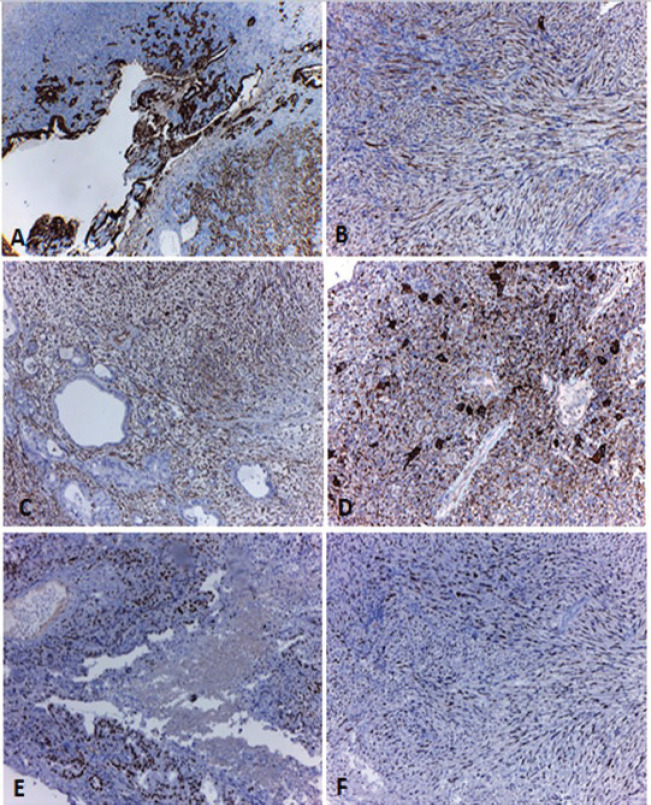
Immunohistochemical techniques. A) Intense positivity with the cytokeratin cocktail (pan keratin) in the epithelioid and spindle cell components of the tumour. B and C) High magnification of the spindle cell component where coexpression of pan keratin (B) and vimentin (C) can be identified. D) Positivity with CD68 in the OGC and the histiocytes. E and F) Overexpression of p53 in the neoplastic epithelial (E) and spindle cell components (F). Original magnification: 40× (A); 100× (B, C, D, E and F). Ref: OGC: osteoclast-like giant cells.

The histological sections of the material embedded in paraffin and coloured with haematoxylin and eosin, showed a neoplastic proliferation constituted by cells with marked atypia, arranged in cords and duct-like structures (Figure 3A and C). In some areas, this lesion acquired a spindle cell pattern with marked pleomorphism (Figure 3B and D). The neoplasia was accompanied by numerous OGC (Figure 3E), extensive areas of necrosis and haemorrhage. An epithelial component of carcinoma *in situ* was also identified. Immunohistochemical techniques were carried out on three micron histological samples using an automated system in accordance with the manufacturer’s guidelines (Benchmark XT, Ventana). Table 1 shows the immunoprofile of the different types of cells of this neoplasia and the monoclonal antibody used. In the epithelioid component, intense positivity was observed with the cytokeratin cocktail and with cytokeratin 7 (Figure 4A). The spindle cell component coexpressed cytokeratins and vimentin (Figure 4B and C) and negativity with smooth muscle actin and S100 protein. The OGC and the histiocytes were positive for CD68 (Figure 4D). The p53 protein was overexpressed in both epithelial and spindle cell neoplastic components, but was negative in the OGC and histiocytes (Figure 4E and F). According to the results of histopathological and immunohistochemical studies obtained during the EUS guided biopsy and polypoid lesion resected endoscopically, the diagnosis of an UC-OGC was established.

## Discussion

UC-OGC is a rare tumour, currently considered to be a variant of PDAC [3, 7].

This tumour is characterised histologically by the presence of a neoplastic epithelial component accompanied by non-neoplastic elements, consisting of OGC and histiocytes [7]. The interest of reporting this case of UC-OGC focuses on different aspects. On one hand, these uncommon tumours account for less than 1% of all pancreatic malignancies. This fact, associated with the precedent that the majority of publications are reports of single cases, makes knowledge of their true frequency inexact [2, 3, 8].

On the other hand, our underlying interest in this case is from an endoscopic ultrasound point of view. Even though the biopsy sample obtained by EUS is limited compared to surgical specimens, in this case, it was useful for histopathological study and tumour staging. Different reports demonstrate the effectiveness of this method in the diagnosis of UC-OGC, as well as the advantage of being less invasive than surgery [1, 9–12]. On a histopathological level, the overlapping descriptions of UC-OGC with other pancreatic giant cell tumours, especially with anaplastic carcinoma of the pancreas (ACP) with pleomorphic bizarre cells, make the distinction between them not always clear. Although, in both tumours, the neoplastic component is constituted of epithelioid and spindle cells with marked atypia, the giant cells are pleomorphic in ACP and reactive in UC-OGC. The differentiation between the two entities is of great importance since they have different prognoses [5, 7, 13–16].

In this case, we identified a proliferation of atypical glands accompanied by a spindle cell component. The multinucleated giant cells throughout the sample showed osteoclast-like morphology without atypia; therefore, the tumour was classified as UC-OGC. As in other series, the expression of cytokeratin in the adenocarcinoma with coexpression of vimentin in the poorly differentiated component supports the idea that the tumour origin is in the duct epithelium and it has areas of mesenchymal differentiation. On the contrary, the negativity with epithelial markers and the positivity with CD68 in the OGC, supports its histiocytic lineage. These cells are considered of a reactive origin since they lack atypia and mutations of K-ras and p53 as observed in the adenocarcinomatous component [5].

The prognosis of UC-OGC is not clear due to the scarce reports of this type of tumour. The behaviour of these neoplasms is unpredictable, with wide ranges of survival rates from a few months, such as in this case, up to 15 years [2–5, 7]. It is postulated that the prognosis of UC-OGC is better than in PDAC and ACP, with a reported mean survival of 7.7 years, compared with PDAC where the mean survival is about 1.6 years [2, 4, 5].

Currently, no standard therapeutic guidelines exist for the management of UC-OGC [3]. Different treatments have been reported, surgical resection being the choice in the early stages. Cases of patients treated with different schemes of adjuvant chemotherapy have also been reported [3, 17–20]. In addition, a case of UC-OGC with liver metastasis treated with radiofrequency ablation and chemotherapy has been published [18]. Finally, based on the experience of radiosensitivity of bone giant cell tumours, the use of radiotherapy has been described as a possible complementary treatment [20, 21]. In our case, the patient was discovered in an advanced stage, with multiple hepatic metastases and significant physical and functional deterioration (Eastern Cooperative Oncology Group 4). Therefore, no specific cancer treatment was commenced, and she died 3 months after initial diagnosis.

## Conclusion

UC-OGC is a rare pancreatic tumour which must be differentiated from PDAC and other neoplasms with giant cells. We present the case of a patient who was not able to undergo surgical treatment due to her advanced stage. However, given the effectiveness of the endoscopic ultrasound-guided procedure and biopsy samples, it was possible to arrive at a diagnosis. We underline the importance of these methods and the multidisciplinary teamwork to deal with complex pancreatic lesions.

## List of abbreviations

- UC-OGC: undifferentiated carcinoma with osteoclast-like giant cells
- OGC: osteoclast-like giant cells
- PDAC: pancreatic ductal adenocarcinoma
- NV: normal value
- EUS: endoscopic ultrasound
- ERCP: endoscopic retrograde cholangiopancreatography
- ACP: anaplasic carcinoma with bizarre pleomorphic cells

## Conflicts of interest

Mariano Villarroel, MD is a Consultant for Boston Scientific. No conflicts of interest have been declared by the other authors.

## Funding declaration

This article was not funded.

## Authors’ contributions

All authors participated in the correction of the text and approved the final version of the document. DS was in charge of the written production. DS, MTD, AI and FV contributed to the diagnostic histology of the case. MV carried out the endoscopic procedures (EUS, ERCP) and edited the video which accompanies the manuscript. MB performed the percutaneous access to the bile duct. AG evaluated the images. LB contributed to the clinical management and treatment of the patient.

## Figures and Tables

**Video 1.**
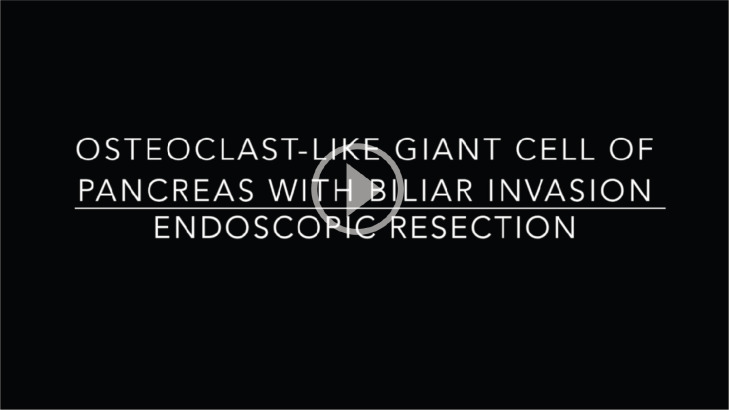
Endoscopic resection: After initial precut a polypoid lesion was identified at the distal common bile duct. It was resected using a polypectomy snare. The specimen was sent to pathology. To view this video, click here https://ecancer.org/journal/14/1072-undifferentiated-carcinoma-with-osteoclastic-giant-cells-of-the-pancreas-diagnosed-by-endoscopic-ultrasound-guided-biopsy.

**Table 1.** Immunohistochemical expression of the different components of the tumour. Ref: (-) negative staining; (+) positive staining. Mm : mouse monoclonal.

Antibodies	Neoplastic mononuclear component		Multinucleated giant cells
	Epithelioid cells	Spindle cells	
Cytokeratin Cocktail (AE1 & AE3;Cell Marque; Mm)	**+**	+	-
Cytokeratin 7 (OV-TL 12/30; Cell Marque; Mm)	+	+	-
Vimentin (V9; Cell Marque; Mm)	-	+	-
Smooth Muscle Actin (1A4; Cell Marque; Mm)	-	-	-
S100 (4C4.9; Cell Marque; Mm)	-	-	-
CD68 (Kp-1; Cell Marque; Mm)	-	**-**	**+**
p53 (DO7; Cell Marque; Mm)	+	**+**	**-**

## References

[1] Reid MD, Muraki T, HooKim K (2017). Cytologic features and clinical implications of undifferentiated carcinoma with osteoclastic giant cells of the pancreas: an analysis of 15 cases. Cancer Cytopathol.

[2] Moore JC, Hilden K, Bentz JS (2009). Osteoclastic and pleomorphic giant cell tumors of the pancreas diagnosed via EUS-guided FNA: unique clinical, endoscopic, and pathologic findings in a series of 5 patients. Gastrointest Endosc.

[3] Moore JC, Bentz JS, Hilden K (2010). Osteoclastic and pleomorphic giant cell tumors of the pancreas: a review of clinical, endoscopic, and pathologic features. World J Gastrointest Endosc.

[4] Muraki T, Reid MD, Basturk O (2016). Undifferentiated carcinoma with osteoclastic giant cells of the pancreas: clinicopathologic analysis of 38 cases highlights a more protracted clinical course than currently appreciated. Am J Surg Pathol.

[5] Jo S (2014). Huge undifferentiated carcinoma of the pancreas with osteoclast-like giant cells. World J Gastroenterol.

[6] Fukushima N, Hruban RH, Kato Y, Bosman FT, Carneiro F, Hruban RH (2010). From ductal adenocarcinoma variants and mixed neoplasms of the pancreas. WHO Classification of Tumours of the Digestive System.

[7] Hruban RH, Adsay NV, Esposito I (2019). From pancreatic ductal adenocarcinoma. WHO Classification of Tumours of the Digestive System.

[8] Abid H, Gnanajothy R (2019). Osteoclast giant cell tumor of pancreas: a case report and literature review. Cureus.

[9] Vilmann P, Jacobsen GK, Henriksen FW (1992). Endoscopic ultrasonography with guided fine needle aspiration biopsy in pancreatic disease. Gastrointest Endosc.

[10] Fujimoto T, Inatomi O, Mizuno R (2018). Anaplastic pancreatic cancer diagnosed with endoscopic ultrasound guided fine needle aspiration showing hypervascular tumor: a case report. Medicine (Baltimore).

[11] Watanabe M, Miura H, Inoue H (1997). Mixed osteoclastic/pleomorphic-type giant cell tumor of the pancreas with ductal adenocarcinoma: histochemical and immunohistochemical study with review of the literature. Pancreas.

[12] Manci EA, Gardner LL, Pollock WJ (1985). Osteoclastic giant cell tumor of the pancreas. Aspiration cytology, light microscopy, and ultrastructure with review of the literature. Diagn Cytopathol.

[13] Mannan R, Khanna M, Bhasin TS (2010). Undifferentiated carcinoma with osteoclast-like giant cell tumor of the pancreas: a discussion of rare entity in comparison with pleomorphic giant cell tumor of the pancreas. Indian J Pathol Microbiol.

[14] Maksymov V, Khalifa MA, Bussey A (2011). Undifferentiated (anaplastic) carcinoma of the pancreas with osteoclast-like giant cells showing various degree of pancreas duct involvement. A case report and literature review. JOP.

[15] Loya AC, Ratnakar KS, Shastry RA (2004). Combined osteoclastic giant cell and pleomorphic giant cell tumor of the pancreas: a rarity. An immunohistochemical analysis and review of the literature. JOP.

[16] Ezenekwe AM, Collins BT, Ponder TB (2005). Mixed osteoclastic/pleomorphic giant cell tumor of the pancreas: a case report. Acta Cytol.

[17] Sah SK, Li Y, Li Y (2015). Undifferentiated carcinoma of the pancreas with osteoclast-like giant cells: a rare case report and review of the literature. Int J Clin Exp Pathol.

[18] Bauditz J, Rudolph B, Wermke W (2006). Osteoclast-like giant cell tumors of the pancreas and liver. World J Gastroenterol.

[19] Wakatsuki T, Irisawa A, Imamura H (2010). Complete response of anaplastic pancreatic carcinoma to paclitaxel treatment selected by chemosensitivity testing. Int J Clin Oncol.

[20] Temesgen WM, Wachtel M, Dissanaike S (2014). Osteoclastic giant cell tumor of the pancreas. Int J Surg Case Rep.

[21] Georgiou GK, Balasi E, Siozopoulou V (2016). Undifferentiated carcinoma of the head of pancreas with osteoclast-like giant cells presenting as a symptomatic cystic mass, following acute pancreatitis: case report and review of the literature. Int J Surg Case Rep.

